# Unilateral anomalous lateral branch of the abdominal aorta piercing the left renal vein – A case report

**DOI:** 10.1590/1677-5449.202401772

**Published:** 2025-10-31

**Authors:** Surekha Devadasa Shetty, Ashwini Aithal Padur, Sapna Marpalli, Satheesha Badagabettu Nayak

**Affiliations:** 1 Division of Anatomy, Department of Basic Medical Sciences, Manipal Academy of Higher Education, Manipal, India.

**Keywords:** abdominal aorta, renal artery, testicular artery, lumbar artery, renal vein, aorta abdominal, artéria renal, artéria testicular, artéria lombar, veia renal

## Abstract

A sound knowledge of variations in the branching pattern of the abdominal aorta is crucial for operative, diagnostic, and endovascular procedures. We report a unilateral anomalous lateral branch of the abdominal aorta observed during dissection classes.This anomalous branch took origin from the anterior surface of the abdominal aorta behind the left renal vein and pierced the left renal vein. After piercing the left renal vein, it traced a tortuous course through the peri-renal fat pad in front of the lower part of the left kidney. The artery then ran downward and laterally, pierced the anterior layer of thoracolumbar fascia, and continued to the posterior abdominal wall to supply its muscles. It also supplied the fat and fasciae around the kidney. Such an anomalous lateral branch could bleed significantly during renal surgery. It could also mislead radiologists during the invasive radiologic procedures.

## Resumo

Um conhecimento sólido das variações no padrão de ramificação da aorta abdominal é crucial para procedimentos cirúrgicos, diagnósticos e endovasculares. Relatamos um ramo lateral anômalo unilateral da aorta abdominal observado durante as aulas de dissecção. O ramo teve origem na superfície anterior da aorta abdominal, atrás da veia renal esquerda, e perfurou a veia renal esquerda. Após perfurar a veia renal esquerda, seguiu um trajeto tortuoso através da camada de gordura perirrenal em frente à parte inferior do rim esquerdo. A artéria corria para baixo e lateralmente; perfurou a camada anterior da fáscia toracolombar e continuou até a parede abdominal posterior para suprir seus músculos. Também supria a gordura e as fáscias ao redor do rim. O ramo lateral anômalo pode sangrar significativamente durante cirurgias renais. Também pode induzir os radiologistas ao erro durante procedimentos radiológicos invasivos.

## INTRODUCTION

The abdominal aorta is the continuation of the thoracic aorta. It extends from vertebrae T12 to L4, where it divides, forming the left and right common iliac arteries. It serves as the main arterial trunk that supplies all the abdominal organs, abdominal walls, lower limbs, and genital organs. The branches of the aorta exhibit a wide range of variations and these variations are well documented in the literature.^1,2^

Renal veins are the major tributaries of the inferior vena cava. The right renal vein drains the venous blood from the right kidney, whereas the left renal vein collects the venous blood from the left suprarenal gland, left gonad, and left kidney. The testicular arteries usually arise from the anterior aspect of the abdominal aorta at the level of the second lumbar vertebra, a little below the origin of the renal arteries. Each of them runs downwards behind the parietal peritoneum before entering the inguinal canal. The right testicular artery crosses the anterior surface of the inferior vena cava. As variants, the testicular artery may originate from the renal artery, suprarenal artery, or lumbar artery.^3^ The testicular arteries are accompanied by the testicular veins. These are formed by the pampiniform plexus, which is formed by the union of small veins from the testis and epididymis. The testicular arteries show variations in 4.7% of people. These variations are mainly described as high origin from the aorta or origin from the renal artery.^4^ This case reports presents a rare anomalous lateral branch of the abdominal aorta. We would like to alert radiologists, nephrologists, and surgeons in general about this artery, which could cause iatrogenic injuries or confusion during radiologic interpretation.

Ethics committee clearance was obtained for this study. It complies with the Helsinki Declaration and with local ethical guidelines. We have obtained all appropriate consent forms and ethics committee clearance for the use of cadavers in this study. No patient data were used in the study.

## CASE REPORT

**Figure 1:**
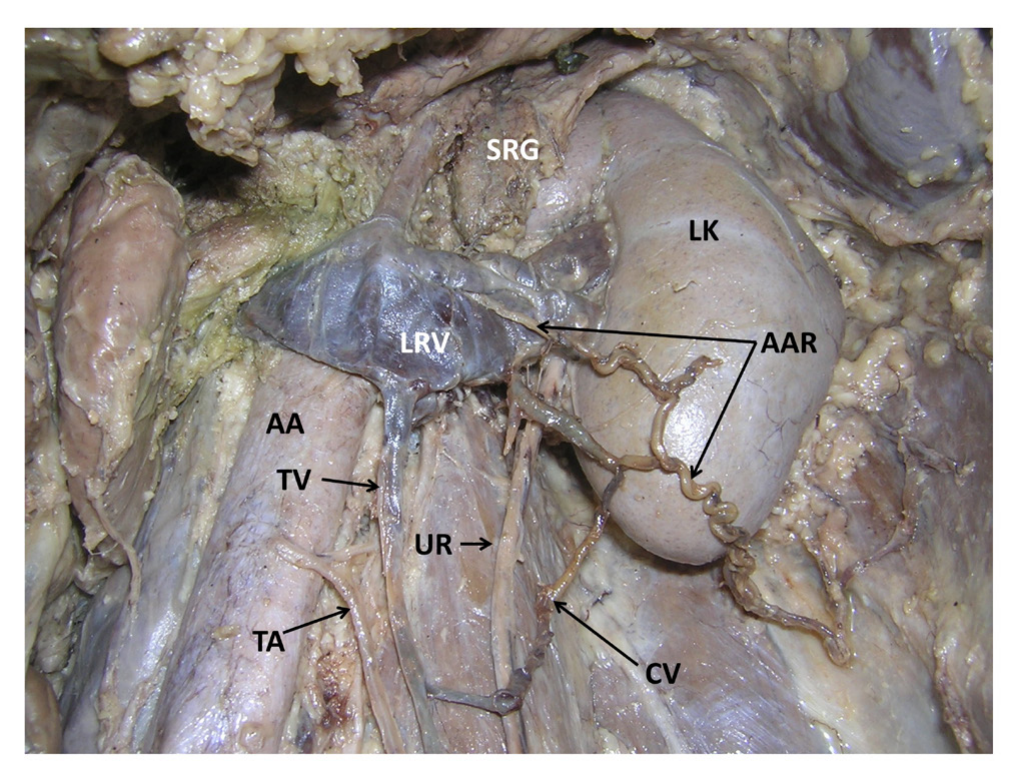
Dissection of the upper left part of the abdomen showing the kidney and its vessels. SRG: left suprarenal gland; LK: left kidney; AAR: anomalous artery; LRV: left renal vein; AA: abdominal aorta; TV: testicular vein; TA: testicular artery; UR: left ureter; CV: communicating vein.

**Figure 2:**
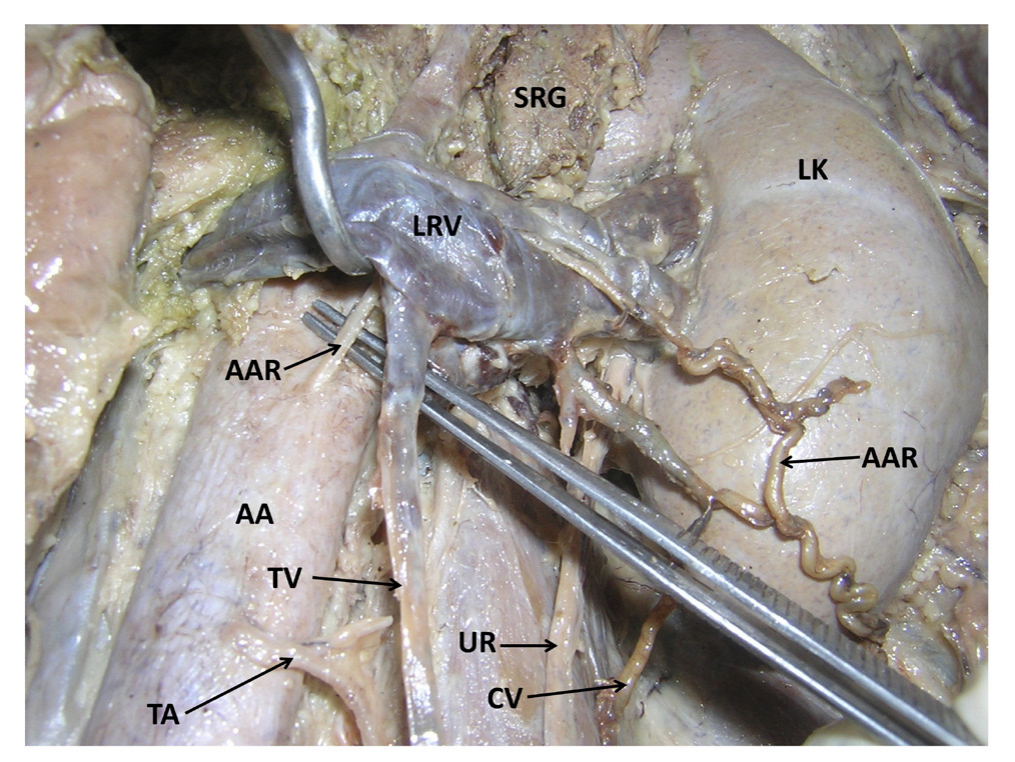
Closer view of the anomalous lateral branch of the abdominal aorta. SRG: left suprarenal gland; LK: left kidney; LRV: left renal vein; AAR: anomalous artery; AA: abdominal aorta; TV: testicular vein; TA: testicular artery; UR: left ureter; CV: communicating vein.

During regular dissection classes, we found some vascular variations in relation to the left kidney in an adult male cadaver aged approximately 70 years. The left kidney was fed by renal vessels which had their normal origin, course, and termination. Moreover, the abdominal aorta gave off almost all of its branches at normal levels. However, an additional, anomalous artery took origin from the anterior surface of the aorta behind the left renal vein and pierced the left renal vein (Figures 1 and 2). After piercing the left renal vein, it traced a tortuous course through the peri-renal fat pad in front of the lower part of the left kidney. The artery then ran downward and laterally, pierced the anterior layer of thoracolumbar fascia, and continued to the posterior abdominal wall to supply its muscles. It also supplied the fat and fasciae around the kidney. This anomalous artery was accompanied by a vein, which opened into the left renal vein close to the hilum of the kidney. There was also a communicating vein, which connected the left testicular vein to the vein accompanying the anonymous artery. The left testicular artery had a slightly low origin from the anterior surface of the aorta, possibly because the anomalous artery took origin from the aorta at the level of the origin of the testicular artery. The upper four lumbar arteries had normal origins from the dorsal surface of the aorta. Their course and distribution were also normal.

## DISCUSSION

Knowledge of variations involving the blood vessels of the abdomen is important for surgeons during operative and endovascular procedures. Variations in the branches of the abdominal aorta are commonly observed during diagnostic angiography, surgeries, or cadaveric dissection. Among these, the most reported involve the hepatic artery, renal artery, celiac trunk and its branches, suprarenal arteries, or gonadal arteries. In the present case, we observed a novel anomalous lateral branch that arose from the abdominal aorta. Previous reports describing anomalous left testicular arteries include a rare case of a left testicular artery getting entrapped between the two divisions of the left renal vein.^5^ The point of origin of the anomalous lateral branch in the current case is almost the same as the above case, but the area of distribution and relationship to the renal veins are totally different.

Variations of the testicular arteries are common and have been well-documented. The testicular arteries may vary at their origin, they may be missing, or one or both arteries may arise from the renal artery, suprarenal artery, or lumbar artery. They may also arise from a common trunk or be doubled, tripled, or quadrupled.^6^ Ravery et al.^7^ reported an anomalous origin and described the surgical importance of a testicular artery emerging from the inferior polar artery of the kidney. A high origin of a testicular artery can be the cause of hemodynamic insufficiency involving kidney and testis. Decreased blood flow may lead to varicocele and testicular atrophy.^8^ Cases of high origin of testicular arteries have been reported previously. However, a low testicular artery origin is seldom reported in the literature. In the present case, the left testicular artery had a slightly low origin from the anterior surface of the aorta.

Various morphological anomalies of the testicular arteries hold tremendous clinical relevance due to their influence on normal functioning of the testis and development of many new surgical techniques within the abdominal cavity.^9^ The gonadal vessels must be preserved to avoid possible complications following damage to these vessels.

Variations of renal veins are common. Bandopadhyay and Saha have reported a case of the left renal vein receiving an additional tributary coming from the posterior abdominal wall. This tributary was hooking the left testicular artery.^10^ It is not uncommon for a lumbar vein to join the left renal vein. In a study by Baniel et al., a lumbar vein ending in the left renal vein was documented in 43% of cases.^11^

Variations involving testicular veins are also common. Incidents of variations of the left testicular vein are more common than those involving the right testicular vein. Asala et al.^12^, reported testicular vein variations in 21.3% of cadavers. In a study conducted by Favorito et al.^13^, variation of the number of right testicular veins was found in 15% of specimens and variations in the number of left testicular veins were observed in 18% of specimens. A case of low origin of the testicular artery with an arteriovenous communication between the left testicular artery and the left testicular vein has also been reported.^14^ Yılmaz et al.^15^ present a case of left renal vein fenestration in which a high origin testicular artery passed through it.

In the current case, an anomalous lateral branch was accompanied by a vein, which opened into the left renal vein. There was also a communicating vein, which connected the left testicular vein to the vein accompanying the anonymous artery.

Embryologically, the testis develops at the thoraco-abdominal junction, retroperitoneally, and later descends to the scrotum. At the same time, the kidney develops at the pelvic cavity and ascends to the upper abdomen. One main difference in the descent of the testis and ascent of the kidney is that the kidney is supplied by different arteries at different levels as it ascends. But the testis is supplied by one artery which is dragged all the way to the scrotum. In the current case, it is possible that the testis also had an artery which was supplying it when it was in the upper part of the abdomen and later was divorced when the testis descended to a lower level. The divorced upper testicular artery later would have crossed the kidney and supplied the abdominal muscles. Its origin and course passing through the renal vein make us think along these lines.

To the best of our knowledge, this is a unique variation, and a similar variation has not been reported previously. Functionally, these vessels might not have much significance as there are lumbar arteries to supply the posterior abdominal wall, but surgically they are of great significance. Knowledge of such cases has great importance in an abdominal operation or invasive arterial procedure. The anomalous lateral branch and the accompanying vein could bleed significantly in renal transplant surgeries or any other procedures in the perirenal area.

## Data Availability

Data not reported or used: “Data sharing does not apply to this article, as no data were generated or analyzed.”
